# Perioperative Pain Management in Hemophilic Patient Undergoing Orthopedic Surgery: A Narrative Review

**DOI:** 10.3390/healthcare12192007

**Published:** 2024-10-08

**Authors:** Antonio Abed Mahagna, Salvatore Annunziata, Camilla Torriani, Eugenio Jannelli, Benedetta Mascia, Alice Montagna, Mario Mosconi, Consalvo Mattia, Gianluigi Pasta

**Affiliations:** 1 Orthopedics and Traumatology Clinic, IRCCS Policlinico San Matteo Foundation, 27100 Pavia, Italy; 2 Division of Anesthesiology, IRCCS Policlinico San Matteo Foundation, 27100 Pavia, Italy; 3 Division of Anesthesiology, Intensive Care and Pain Medicine, ICOT Polo Pontino, Sapienza University of Rome, 04100 Rome, Italy

**Keywords:** hemophilia, pain, orthopedic surgery

## Abstract

Background: Hemophilia type A and B is associated with spontaneous bleeding in muscle tissues and joints. Acute hemarthrosis, representing 70–80% of all bleedings in severe hemophilia patients, is extremely painful. When surgical procedures are needed in hemophiliac patients, perioperative management should be planned with a multidisciplinary team. Our narrative review, through a rigorous analysis of the current literature, focuses on pain management in hemophiliac patients. Methods: The report synthesizes a literature review on hemophilia, adapting PRISMA guidelines. It identifies a research question on surgical procedures and perioperative pain management. Various sources, including electronic databases, are utilized. Study inclusion criteria are defined based on the research question. Forty studies are included. A detailed study selection is illustrated. Results: Guidelines for managing acute postoperative pain in the general population advocate for a multimodal analgesic administration to enhance synergistic benefits, reduce opioid requirements, and minimize side effects. Recent recommendations from the World Federation of Hemophilia (WFH) for postoperative pain management in hemophilia patients suggest tailoring treatment based on pain levels, in coordination with anesthesiologists. Conclusions: Pain management in hemophiliac patients undergoing orthopedic interventions requires a multidisciplinary approach, with further research needed to define a reliable global standard of treatment.

## 1. Introduction

Hemophilia A and B type are associated with spontaneous bleeding in muscle tissues and joints. Acute hemarthrosis, which accounts for 70–80% of all bleeding episodes in patients with severe hemophilia, is extremely painful; repeated joint bleeds predisposed to a vicious cycle of bleeding, synovitis, and more bleeding [1,2,3]. As well as “short-term” effects such as swelling and acute pain, the most serious and challenging to treat are the “long-term” effects. The episodes of hemarthrosis cause joint damage as a consequence of this catabolic activation of the synovium, which results in cartilage damage and hemophilic arthropathy with a development of chronic pain and a reduced quality of life [4,5]. Moreover, joint pain due to bleeding in hemophilia A and B patients is often multi-articular [6]. The damage of the spontaneous hemarthrosis affects shoulders, elbows, hips, knees, and ankles with consequent hypertrophic growth of the epiphysis and deformities in children leading to articular destruction in adults [7]. Nowadays, clotting factor concentrate prophylaxis has been widely used to prevent this progressive articular damage [8]; however, orthopedic surgery procedures are often still required to solve the patients’ needs related to both pain and articular dysfunction. When surgical interventions are required in patients affected by hemophilia, the peri-operative management should be planned with a multidisciplinary team that comprises not only the orthopedic surgeon, but also hematologists, anesthetists, and physiotherapists in order to establish the best approach that combines surgical and medical expertise [9,10].

The objective of this study is to conduct a thorough narrative review of the existing literature on perioperative pain management in hemophiliac patients undergoing orthopedic surgery, evaluating the effectiveness and safety of various and multidisciplinary pain management strategies.

## 2. Materials and Methods

This report provides an account of the review of the available literature conducted to achieve the aim of this research. Its reporting method is an adaptation of the Preferred Reporting Items for Systematic Reviews and Meta-Analyses (PRISMA) guidelines [http://www.prisma-statement.org/, (accessed on 1 October 2023)].

- Identifying the research question:
- The research question identified for the literature review was first of all, to summarize the current knowledge on the surgical interventions required in patients with hemophilia and the need to plan pain perioperative management with a multidisciplinary team.
- Identifying relevant studies:
- A literature search was conducted in order to find all relevant studies on the topic. These were identified by means of diverse sources.
- Electronic database search:

The following electronic databases were searched, taking into consideration the chronological span between 1993 and 1 December 2023: PubMed and Embase. The research strategy was designed to retrieve the most relevant results. Due to the specificity of the two databases employed, for each one, a different search string was built (1: PubMed search string; 2: Embase search string). In brackets, the number of the results is provided).

1. ((((“surgical procedures, operative”MeSH Terms] OR (“surgical”; [All Fields] AND

“procedures“ [All Fields] AND “operative“ [All Fields]) OR “operative surgical

procedures“ [All Fields] OR “surgical“ [All Fields] OR “surgically“ [All Fields] OR

“surgicals“ [All Fields]) AND (“pain“ [MeSH Terms] OR “pain“ [All Fields])) OR

((“perioperative“ [All Fields] OR “perioperatively“ [All Fields]) AND (“manage“ [All

Fields] OR “managed“ [All Fields] OR “management “ [All Fields] OR

“managements“ [All Fields] OR “manager“ [All Fields] OR “manager s“ [All Fields] OR

“managers“ [All Fields] OR “manages“ [All Fields] OR “managing“ [All Fields] OR

“management“ [All Fields] OR “organization and administration“ [MeSH Terms] OR

(“organization“ [All Fields] AND “administration“ [All Fields]) OR “organization and

administration“ [All Fields] OR “management“ [All Fields] OR “disease

management“ [MeSH Terms] OR (“disease“ [All Fields] AND “management“ [All

Fields]) OR “disease management“ [All Fields]))) AND (“haemophilia“ [All Fields] OR

“hemophilia a“ [MeSH Terms] OR “hemophilia a“;[All Fields] OR “hemophilia“ [All

Fields] OR “haemophilias“ [All Fields] OR “hemophilias“ [All Fields]) AND

1993/01/01:2023/12/01[Date—Publication] AND “english“ [Language]) AND

((1993/1/1:2023/12/1[pdat]) AND (english[Filter])) [596];

2. (“surgical pain” OR (surgical AND (“pain”/exp OR pain)) OR (perioperative AND

management)) AND haemophilia AND [english]/lim AND [01-01-1993]/sd NOT [01-

12-2023]/sd [682].

**Figure 1:**
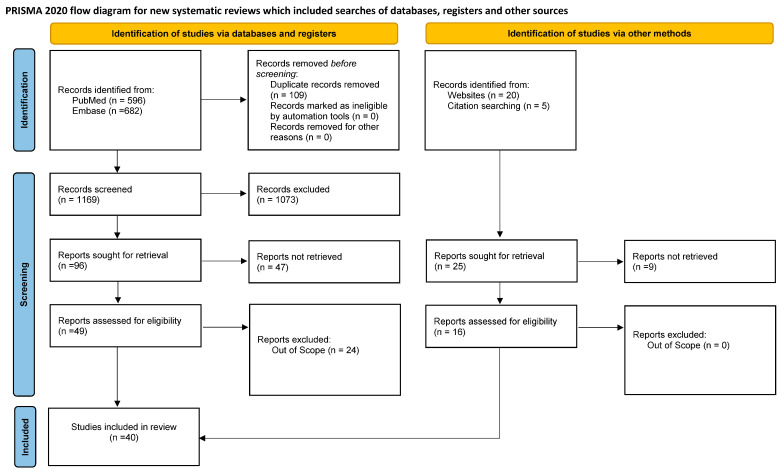
Prisma 2020 flow diagram for new systematic reviews, which included searches of databases, registers, and other sources.

- Other sources:
  Eleven studies were also included, starting from a website and citation research. These articles were regarded to be relevant, even though they were not identified through the search strings.
- Study inclusion criteria:
  Starting from the research question, inclusion and exclusion criteria for the objective selection of the identified studies were defined. Only studies published in the English language between 1993 and 1 December 2023 were eligible for inclusion. Titles and abstracts and full texts were screened by the research team—i.e., two authors performed the study selection and the data extraction independently, and all disagreements were discussed between the authors.
- Data extraction:
  A standardized data extraction sheet was prepared, where main information on the studies was collected (e.g., first author’s name, study title, publication year, and DOI).
- Study selection:
  Via the literature search, thirty-six studies were included in this literature review; twenty-five of them were identified via database searches and sixteen via websites or citation searching (Figure 1). Figure 1 shows the process of study selection in detail, covering the number of search records retrieved from the two database searches (*n* = 1278) and all other searches (*n* = 25), the number of screened titles/abstracts (*n* = 1169), and the number of finally included studies (*n* = 40).

## 3. Results

### 3.1. Pain Assessment

The Multidimensional Hemophilia Pain Questionnaire (MHPQ) is a valid tool for discerning the type of pain, investigating the situations that most sensitize the patient’s pain perception according to the 0–10 scale (where 0 is no pain and 10 is the worst pain possible), thus providing a more accurate guide in the therapeutic choice.

### 3.2. Pharmacological Pain Approach

#### 3.2.1. Topical Analgesics for Hemophilia Patients

Indications for topical analgesics include musculoskeletal pain, ligament injuries, joint diseases, muscle pain syndrome, and various spine-related pains. The analgesic effect of topical NSAIDs corresponds to about 50% of the effect reached via systemic administration and entails significantly fewer adverse effects [11].

#### 3.2.2. Nonsteroidal Anti-Inflammatory Drugs and Coxibs

Both NSAIDs and selective COX-2 inhibitors (coxibs), target the cyclooxygenase enzyme and have analgesic, antipyretic, and anti-inflammatory effects. There are two isoenzymes: COX-1 and COX-2 [12,13]. COX-1 protects the stomach lining, regulates renal perfusion, and induces platelet aggregation via the production of thromboxane A2 in thrombocytes. In inflammation or swelling, the net analgesic effect of this drug class can be superior to that of opioids. This goes for, e.g., naproxen, ibuprofen, mefenamic acid, and diclofenac [14]; however, significant adverse effects are possible. Among this drug class, acetylsalicylic acid (ASA) causes the most problems; even in small doses (30–50 mg), ASA irreversibly blocks COX-1 in platelets, thus negatively influencing coagulation [15]. Consequently, ASA is thoroughly unsuited for treating pain in hemophilia patients.

#### 3.2.3. Paracetamol

It is indicated for mild to moderate levels of pain. It has an antipyretic but no anti- inflammatory effect and has been historically proposed to selectively inhibit COX-2. According to available studies [16], paracetamol seems to have in vitro screens a low potential on the inhibition of COX-1 by inhibiting an enzyme variant of COX-1 and enhancing the serotonergic pathway. The recommended dosage to be administered at any one time is 10–15 mg/kg body weight. When overdosed multiple times, paracetamol has a hepatotoxic effect, and so the approved maximum individual dosage of 15 mg/kg of body weight or maximum individual dosage of 1 g and maximum daily dosage of 4 g for people weighing >50 kg should never be exceeded [17]. Overdosing can lead to liver failure. Paracetamol does not inhibit platelet aggregation. Due to the peripheral selective COX-2 inhibition, prolonged paracetamol administration increases the cardiovascular risk.

#### 3.2.4. Dipyrone (Metamizole)

Metamizole (World Health Organization [WHO] name)/dipyrone (American and British name) is a prostaglandin synthetase inhibitor that inhibits COX-1 and COX-2. In contrast to other nonselective NSAIDs, it has been proven for a long time that it causes fewer ulcers and less bleeding in the upper gastrointestinal tract [18], thus making it safer in hemophiliac patients. Moreover, the recent published literature does not mention renal impairment as an adverse effect of dipyrone (metamizole) [19]. The temporary platelet dysfunction caused by dipyrone (metamizole) remains controversial and subject to criticism on the methodologies adopted in reporting adverse events in registries [20,21].

#### 3.2.5. Opioids

Opioids are generally used in combination with two non-opioid drugs, to maximize the analgesic effect by reducing the minimum effective dose [22]. Opioids are commonly prescribed for more severe chronic pain. [23].

#### 3.2.6. Regional Analgesia

Neuraxial techniques are contraindicated in hemophilia for the risk of spinal/epidural hematoma. The use of peripheral regional analgesia (i.e., nerve blocks, field blocks) is poorly described in the scientific literature. The risk of muscle and soft tissue bleeding after peripheral anesthesia is unknown.

### 3.3. Postoperative Pain Treatment

For the postoperative treatment of patients with hemophilia, recent WFH guidelines recommend management proportionate to the level of postoperative pain, in coordination with the anesthesiologist. Specifically, in Recommendation 2.6.5, for patients with hemophilia and postoperative pain, the WFH recommends analgesia similar to that used in patients without hemophilia, including as appropriate, the use of intravenous morphine or other narcotic analgesics, followed by an oral opioid (e.g., tramadol, codeine, hydrocodone, etc.) and paracetamol/acetaminophen as pain decreases. REMARK: With the exception of selective COX-2 inhibitors, NSAIDs should not be used in patients with hemophilia [24].

Even in patients with hemophilia, intravenous opioids therefore remain fundamental for the treatment of acute postoperative pain of high intensity, and their administration must be adapted to the individual patient, also considering his level of preoperative pain, carrying out pain monitoring with validated scales during the postoperative period, so as to be able to individualize the administration of analgesics [22].

A combination of opioids with acetaminophen may allow doses to decrease and be discontinued rapidly in the days following surgery, thus reducing the risk of their prolonged and inappropriate use [25].

## 4. Discussion

Although the widespread use of factor VIII/IX replacement therapy has significantly reduced the severity of arthropathy, pain in one or more joints is a daily reality for as many as two-thirds of patients with severe hemophilia and leads patients to ask for orthopedic interventions [2]. Pain is therefore a critical aspect of hemophilia [2] and adds to the burden of the disease. Effective pain management is essential to improve patient outcomes and quality of life; however, the selection and application of appropriate pain-relieving therapies and the perioperative pain management in case of surgery remain a challenge for the anesthesiologist [4]. Orthopedic surgery is indicated in cases of severe muscle stiffness, disabling bone deformities and advanced arthropathies, especially when concerning the knee, as it is considered the standard procedure in end-stage hemophilic knee arthropathy [26].

Although MHPQ is a widely validated questionnaire [27], it is necessary to consider the presence of “confounding factors” that can alter the reliability of the answers provided by the patient, such as feelings of bewilderment and depression caused by long-standing pain, causing higher scores to be provided. Moreover, the presence of moderate or severe cognitive impairment requires the use of simplified tests such as face pain scales or specific tools like PAINAD test (pain assessment in advanced dementia) [28].

The clinical evidence for the effectiveness of topical NSAIDs is generally quite convincing, particularly when it comes to acute musculoskeletal pain syndromes [11]. Dermal tolerability of topical NSAIDs is good.

COX-2 inhibitors, such as celecoxib, are better suited for pain therapy in hemophilia patients due to their lower risk of gastrointestinal adverse effects. Etoricoxib was investigated in patients older than 12 years (*n* = 102) and proved to be effective, safe, and welltolerated when administered to patients with arthropathy [29]. Thanks to the well-known renal, cardiovascular, and gastrointestinal safety profile, coxibs as well as NSAIDs should only be prescribed in the lowest effective dosage and for as short a time as possible. In cases of an existing cardiovascular comorbidity, the COX-2 inhibition leads to an exponential risk of recurrent cardiovascular events [30]. Severe cardiovascular underlying diseases and cardiac insufficiency are therefore to be regarded as contraindications for COX-2 inhibitors.

Regarding dipyrone, case-control studies with large sample sizes have shown an incidence of agranulocytosis less than one case in a million [31,32,33]. Consequently, the use of short-term dipyrone (metamizole) is justifiable even in hemophiliac patients. In a postoperative setting and in case of severe trauma, since NSAIDS use is associated with a negative impact on bone healing [34] and there is an increased risk of associated bleeding, factor substitution and balancing coagulation in hemophiliac patients is paramount. If dipyrone (metamizole) is used, a careful analysis of the blood count profile and an evaluation adapted to the individual case is recommended.

The current literature shows opioids are more effective for short-term pain relief than a long-term one, with variable risks of dependence in chronic use, especially with more potent opioids [35]. If their use is already widely confirmed in cancer pain, a careful case-by-case analysis should be reserved in the other scenarios [3], remaining in most cases a second choice after the use of paracetamol and NSAIDs.

About regional analgesia, some authors reported a case of penile nerve block in urinary surgery in hemophilic patients with good results [36]; in our experience, after an infusion of clotting factors, the use of an ultrasound-guided block for superficial nerves or nerve-fields (such as femoral nerve, iliac fascia) has shown to be effective (excellent pain control both at rest and during exercise) and not associated with additional regional bleeding; thus, for fragile patients with multiple comorbidities in which almost every pharmacological intervention is contraindicated, the practice of regional analgesia could be an optimal chance to manage postoperative pain. Further studies are necessary to establish whether these techniques are completely safe.

Further research is needed to find a reliable and worldwide reproducible gold standard of treatment since the current guidelines for the management of acute postoperative pain in general population merely recommend the administration of analgesics in multimodal combination, to facilitate synergistic benefit, reduce opioid requirements, and reduce side effects [37].

### Quality Assessment and Risk of Bias

In accordance with the nature of this narrative review, a formal quality assessment of the included studies was not performed using standardized tools such as the Cochrane Risk of Bias (RoB) tool or the Newcastle-Ottawa Scale. However, we recognize the need to critically appraise the quality and potential biases of the literature examined.

The studies included in this review span a variety of research designs, from randomized controlled trials to observational studies and case reports, which inherently introduces variability in study quality. For instance, several studies lacked sufficient methodological details, particularly regarding randomization and blinding procedures, which raises concerns about selection bias and performance bias.

Moreover, some studies did not provide complete follow-up data on the patients involved. This is evident in studies evaluating surgical interventions and postoperative pain management, where the long-term follow-up and comprehensive reporting of patient outcomes are crucial to understanding the effectiveness and safety of these approaches.

Although efforts were made to include non-indexed sources and gray literature, the reliance on published data may have led to an underrepresentation of studies with negative or null results. This could affect the overall synthesis of evidence, particularly in areas such as postoperative pain management, where negative findings might be underreported.

Lastly, the heterogeneity in study designs, populations, and outcomes presents challenges in drawing direct comparisons across studies, especially in sections discussing the effectiveness of pharmacological treatments and regional analgesia. The variability in methodologies complicates the assessment of generalizability and increases the risk of confounding factors that could influence the reported outcomes.

## 5. Conclusions

Although pain management is a critical issue for the hemophilic patient undergoing orthopedic surgery, very little of the literature is available on this topic [38,39]. The importance of a multidisciplinary approach must be stressed to provide the best care and to reach the most effective patient-specific decisions [40].

## Data Availability

No new data have been created.
